# Imbalance between default mode and sensorimotor connectivity is associated with perseverative thinking in obsessive-compulsive disorder

**DOI:** 10.1038/s41398-022-01780-w

**Published:** 2022-01-12

**Authors:** Emily R. Stern, Goi Khia Eng, Alessandro S. De Nadai, Dan V. Iosifescu, Russell H. Tobe, Katherine A. Collins

**Affiliations:** 1 Department of Psychiatry, New York University Grossman School of Medicine, New York, NY USA; 2 Nathan S. Kline Institute for Psychiatric Research, Orangeburg, NY USA; 3 Department of Psychology, Texas State University, San Marcos, TX USA

**Keywords:** Psychiatric disorders, Neuroscience

## Abstract

Obsessive-compulsive disorder (OCD) is highly heterogeneous. Although perseverative negative thinking (PT) is a feature of OCD, little is known about its neural mechanisms or relationship to clinical heterogeneity in the disorder. In a sample of 85 OCD patients, we investigated the relationships between self-reported PT, clinical symptom subtypes, and resting-state functional connectivity measures of local and global connectivity. Results indicated that PT scores were highly variable within the OCD sample, with greater PT relating to higher severity of the “unacceptable thoughts” symptom dimension. PT was positively related to local connectivity in subgenual anterior cingulate cortex (ACC), pregenual ACC, and the temporal poles—areas that are part of, or closely linked to, the default mode network (DMN)—and negatively related to local connectivity in sensorimotor cortex. While the majority of patients showed higher local connectivity strengths in sensorimotor compared to DMN regions, OCD patients with higher PT scores had less of an imbalance between sensorimotor and DMN connectivity than those with lower PT scores, with healthy controls exhibiting an intermediate pattern. Clinically, this imbalance was related to both the “unacceptable thoughts” and “symmetry/not-just-right-experiences” symptom dimensions, but in opposite directions. These effects remained significant after accounting for variance related to psychiatric comorbidity and medication use in the OCD sample, and no significant relationships were found between PT and global connectivity. These data indicate that PT is related to symptom and neural variability in OCD. Future work may wish to target this circuity when developing personalized interventions for patients with these symptoms.

## Introduction

Obsessive-compulsive disorder (OCD) is characterized by the presence of obsessions, defined as thoughts, urges, or images that cause anxiety and are experienced as intrusive and inappropriate, and/or compulsions, which are repetitive behaviors typically performed to relieve anxiety caused by obsessions. While the diagnostic criteria for OCD are fairly straightforward—requiring the presence of obsessions or compulsions with distress or functional impairment—the phenotypic presentation of the disorder is actually highly heterogeneous. Factor analytic studies examining the content of OCD symptoms [1–4] typically reveal between 3 and 4 main factors or dimensions including: contamination/cleaning symptoms; concerns about being responsible for causing harm or bad outcomes with checking compulsions; symmetry/ordering/arranging symptoms driven by the sensation or perception that something is “not just right” (“not-just-right-experiences” [NJREs]) or “incomplete”—symptoms that are sometimes referred to as “sensory phenomena” [5–8]; and repetitive thoughts related to sexual, religious, or violent themes, which are experienced as disturbing and highly ego-dystonic (“unacceptable thoughts”). Although a given patient often experiences symptoms associated with more than one factor, cluster analyses have identified clinical symptom subgroups in OCD that are characterized by a preponderance of symptoms in one factor over others [9–13].

Obsessions have been characterized as an OCD-specific instantiation of a perseverative thinking style that is prevalent across internalizing disorders [14]. Generally speaking, perseverative negative thought (PT) describes a repetitive thought process that is characterized by intrusive and uncontrollable negative thoughts about the self and current, past, or future events [15–17]. PT is transdiagnostic—being prevalent in several disorders including generalized anxiety and major depression [14, 15, 18–22]. Conceptualized as an inflexible and therefore maladaptive pattern of spontaneous thought that exists on a spectrum with mind-wandering [23], PT has been proposed to result from a difficulty in interpreting internal body signals [24]. Brosschot et al. [25] and Ottaviani et al. [23, 26] theorize that PT engenders persistent emotional and physiological activation in preparation for defensive action that has detrimental effects on psychiatric and physical health similar to those associated with real-life exposure to chronic stress. Indeed, PT is associated with somatic complaints [25, 27, 28] and has been linked to changes in cardiac, hypothalamic-pituitary-adrenal (HPA) axis, and immune system functioning [25]. Like chronic stress, PT has also been shown to play a role in the development and maintenance of psychopathology. PT predicts severity of future depression and anxiety when including baseline levels of these symptoms in analytic models [21, 22] and mediates the effects of negative cognitive style, self-criticism, and maladaptive perfectionism on severity of depression and anxiety [15, 29, 30].

Despite its known importance for internalizing psychopathology, PT has not been extensively investigated in OCD. One prior study using the transdiagnostic, content-independent perseverative thinking questionnaire (PTQ [16]) found that PT is higher in OCD patients than healthy controls [17]. Although this study identified increased PT among OCD patients, it is unclear whether all dimensions or types of OCD are associated with elevated PT, or whether PT is linked to only certain types of symptoms of the disorder. Raines and colleagues [31] found a correlation between PT and “unacceptable thoughts” OC symptoms in a transdiagnostic patient sample. However, the sample in this study was composed of treatment-seeking individuals that primarily carried diagnoses of depression or generalized anxiety, and only 6% had a primary or secondary diagnosis of OCD. In addition, PT was measured with the Ruminative Response Scale (RRS), which measures the tendency to repetitively think about the causes and consequences of low or sad mood, thus being tailored to the study of rumination in depression [32] more than to OCD. Finally, in an investigation examining PT in a non-clinical undergraduate sample [33], overall OC severity and various subscales of Padua Inventory-Revised [34] (e.g., “washing”, “checking” and “precision”) were positively related to scores on the RRS, even after accounting for variance associated with depression. Despite these intriguing findings, interpretation of these results is limited by the relatively small proportion of patients who had a clinical diagnosis of OCD as well as the reliance on the depression-focused RRS scale as the primary measure of PT. As such, further research is needed to examine the relationships between PT and symptom dimensions in OCD patients.

To our knowledge, no studies to date have investigated the relationship between brain function and PT in OCD. A recent meta-analysis [35] examined perseverative cognition—defined broadly using the RRS, Penn State Worry Questionnaire, and other self-report and task-related behavioral measures of PT—in relation to task-activation and resting-state functional connectivity in healthy individuals and a transdiagnostic sample of clinical patients. Results revealed that multiple regions of the default mode network (DMN), including ventral/anterior medial prefrontal cortex (including pregenual anterior cingulate cortex [ACC] and medial frontal pole areas), posterior cingulate cortex (PCC)/precuneus, medial (parahippocampal gyrus) and lateral (middle temporal gyrus) temporal cortex, were associated with perseverative cognition across the full sample [35]. The DMN is a well-delineated brain system that is reduced in activity when attention is directed to the external environment, but becomes active when individuals engage in self-focused internal mentation related to thinking about the self, autobiographical memory, and thinking about the future [36–38], i.e., those processes typically invoked during PT. The majority of clinical patients included in the meta-analysis were those with major depression and generalized anxiety, and only one of the studies was conducted in OCD. This study, however, did not examine the relationship between individual variability of PT and brain function but instead focused on task-related comparisons aiming to evoke internally-directed perseverative thinking averaged across the OCD sample [39]. It thus remains unclear whether variability of PT is linked to DMN functioning in OCD, similar to the multiple reports linking DMN to rumination in depression [40–47]; (see [48, 49] for reviews), and whether PT-related circuitry is associated with clinical heterogeneity of OC symptoms.

The present study addressed these questions by investigating variability of perseverative negative thinking in relation to resting-state functional connectivity using magnetic resonance imaging (rs-fcMRI) in a sample of OCD patients. Resting-state functional connectivity measures correlations between low-frequency blood oxygen level dependent (BOLD) fluctuations, which identify “intrinsic” (i.e., task-independent) networks in the brain that are thought to reflect polysynaptic communication between diverse areas of the brain [50]. Although rs-fcMRI networks are modulated by cognitive context [51–53], they tend to be more stable across time than task-related activations [51, 54]. Prior work has highlighted the promise of investigating intrinsic connectivity patterns in psychiatric disorders to identify novel and personalized treatment targets [55, 56]. In the present study, we measured the association between scores on the transdiagnostic perseverative thinking questionnaire (PTQ [16]) and whole-brain rs-fcMRI measures of local and global connectivity [57], and interrogated the relationship between PT-related connectivity and OC symptom dimensions. To aid in interpretation of PT-related connectivity patterns in OCD, we also compared rs-fcMRI patterns in “high”- and “low”-PTQ OCD patients with that observed in an independent sample of healthy controls.

## Materials and methods

### Subjects and procedure

Subjects were recruited at the Icahn School of Medicine at Mount Sinai (ISMMS), Nathan Kline Institute for Psychiatric Research (NKI), and New York University School of Medicine (NYUSoM) between May 2014 and March 2020. Ninety patients with OCD participated within that time frame (17 were recruited at ISMMS, 19 at NKI, and 54 at NYUSoM). Fifty-four healthy controls also completed the study (39 were recruited at ISMMS, 7 at NKI, and 8 at NYUSoM). ISMMS-recruited subjects were scanned at ISMMS, and NYUSoM- and NKI-recruited participants were scanned at NKI. Data from 5 OCD patients and 3 controls were excluded (4 patients were excluded for having high motion requiring censoring [58] [see below for detail]; 1 patient met study exclusion criteria after questionnaire completion; 3 controls had missing data on the PTQ). Final data were analyzed from 85 OCD patients (17 were recruited at ISMMS, 17 at NKI, and 51 at NYUSoM) and 51 controls (36 were recruited at ISMMS, 7 at NKI, and 8 at NYUSoM). The study protocol was approved by the Institutional Review Boards at each institution and all subjects provided written informed consent.

All patients met DSM-5 criteria for OCD and were excluded for lifetime history of bipolar and psychotic disorders or current moderate/severe alcohol or substance use disorders. Diagnoses were made by a trained rater using the Mini International Neuropsychiatric Interview [59]. Among the 85 patients, 57 (67%) had at least one current comorbid Axis I disorder, and 44 (52%) were taking psychotropic medications. Healthy controls were free of current and past Axis I diagnoses and psychotropic medications. See “Supplementary Materials” for further details on comorbidities and medication for the patient sample.

### Behavioral and clinical assessments

Perseverative negative thinking was measured using the PTQ [16], which is a 15-item scale that assesses thought repetitiveness and intrusiveness, and difficulty with thought disengagement, independent of specific content. Example statements are: “The same thoughts keep going through my mind again and again” and “My thoughts prevent me from focusing on other things”. Responses on 5-point Likert scales indicate the frequency with which a subject has similar experiences (0 = never to 4 = almost always). The PTQ shows high internal consistency and convergent validity with other measures of repetitive thought [16]. Each subject’s total PTQ score was computed as the average of their responses across all items (0 = no PT, 4 = extreme PT).

OCD symptoms were measured using the clinician-administered Yale-Brown Obsessive Compulsive Scale (Y-BOCS [60]). OCD symptom subtypes were evaluated with the self-report Dimensional Obsessive-Compulsive Scale (DOCS [3]), which assesses severity of OCD symptoms in four categories: (1) concerns about germs and contamination (“contamination”); (2) concerns about responsibility for harm, injury, or bad luck (“responsibility for harm”); (3) unacceptable or taboo thoughts (e.g., about sex, immorality, or violence) (“unacceptable thoughts”); and (4) concerns about symmetry, completeness, and the need for things to be “just right” (“symmetry/NJRE”). State anxiety and depression were measured using the Beck Anxiety Inventory (BAI [61]) and the Quick Inventory for Depression Symptomatology (QIDS [62]), respectively.

### Neuroimaging data acquisition and preprocessing

Details on MRI data acquisition parameters and data preprocessing are provided in the supplemental materials. MRI scanning occurred on Siemens 3 T scanners (ISMMS-recruited subjects were scanned on a MAGNETOM Skyra and NYUSoM- and NKI-recruited subjects were scanned on a MAGNETOM TrioTim), with sequences harmonized between the two scanning sites. In addition to sequence harmonization, we further addressed potential effects of site-based clustering in measurements by including site as a covariate for all group-level imaging analyses, an approach for clustering recommended by McNeish and Kelley [63] that has also been used by multicenter neuroimaging studies such as the NIMH-funded Adolescent Brain Cognitive Development consortium [64, 65]. For the primary analyses looking at the relationships between PTQ score and brain connectivity in the OCD patient group alone, a 2-level variable for site/scanner (Mount Sinai vs. NKI) was used as the site covariate. Although all HC recruited at Mount Sinai (36 out of 51 subjects) were scanned on the same MRI scanner, two sequences with minor differences were used (one sequence was used for subjects scanned before 2017 and another was used for subjects scanned after 2017; see “Supplementary Materials” for details). For the analyses comparing OCD patients to HC, two dummy variables were used for the 3 sites (Mount Sinai before 2017, Mount Sinai after 2017, NKI).

All participants underwent an eight-minute resting-state scan (480 brain volumes with a TR = 1 s) with eyes opened. Preprocessing was performed using a combination of Statistical Parametric Mapping v.12, scripts taken from the Human Connectome Project preprocessing pipeline [66, 67], AFNI (v.10.6, “3dSkullStrip”), and FSL v.5.0.10. Structural images were skull-stripped, nonlinearly corrected for gradient field distortion, and normalized to an MNI template (the “tissue probability map” [tpm] image in SPM v.12). Preprocessing for functional images included gradient nonlinearity distortion correction, realignment to the first volume of the run, normalization to MNI template, and spatial smoothing using a 6 mm kernel. Six rigid-body realignment parameters (3 for translation: X, Y, and Z; and 3 for rotation: pitch, roll, yaw) were produced for each subject following the realignment step. Registrations of T1-weighted and BOLD images to the MNI template were checked manually for each participant as part of our quality control procedures.

The preprocessed structural and functional data were entered into CONN-fMRI Functional Connectivity Toolbox for SPM (v17f) [57] for further preprocessing before conducting functional connectivity analyses. Physiological noise and other artifacts were removed from BOLD data using component-based noise correction (CompCor) that regressed out the first 5 component timeseries of segmented and normalized cerebrospinal fluid (CSF) and white matter (WM), 12 motion regressors (6 realignment parameters and their first derivatives), and 2 regressors for the “effect of rest” (a function convolved with the hemodynamic response function and its first derivative). The “effect of rest” regressors were included to account for variance related to the ramping up of scanner magnetization as it achieves steady state. Although the MRI scanners acquiring this data already discarded the first 10 volumes in each run, in an abundance of caution we also modeled a further ramping up for ~25 s prior to signal stabilization to account for any persisting effects. Note that the main analyses were repeated without these “effects of rest” regressors and the findings were unchanged. In addition to the denoising described above, high-motion volumes (framewise displacement > 0.5 mm or global signal *Z* > 3) were identified using the Artifact Rejection Toolbox (ART, www.nitrc.org/projects/artifact_detect/) and specified as regressors in the model (one regressor per high-motion volume, as implemented in CONN). Prior work suggests that this type of motion censoring—known as spike regression—combined with other forms of denoising effectively reduces spurious motion-related correlations [68]. Four patients were excluded for having greater than 180 (out of 480) high-motion volumes, consistent with recommendations for the inclusion of at least 5 min (300 volumes with TR = 1 s) of useable resting-state data [58]. BOLD data were band-pass filtered at 0.008 < f < 0.09 Hz. All measures were z-transformed at the individual subject level (within-subject normalization) and statistical testing conducted on these standardized values (see “Supplementary” for a discussion of the use of within-subject normalization).

### Data analysis

#### Functional connectivity measures

Two whole-brain functional brain measures were examined with CONN. First, “integrated local correlation” measures the average correlation between the timecourse of a given voxel and its nearest neighbors [57, 69], with greater local correlation thought to reflect increased local coherence or connectivity of an area. Local correlation is similar to the measure of regional homogeneity (“ReHo” [70, 71]), with several noted advantages [69]. Second, “global correlation” measures the average correlation between the timecourse of a given voxel and every other voxel in the brain [57], with greater global correlation thought to reflect greater “hubness” or interconnectedness of an area with other areas of the brain, akin to a measure of degree centrality [72].

#### Analysis of relationships with PTQ

Within OCD patients, group-level analyses used separate multiple regressions to examine the relationship between PTQ (independent variable) and brain measures (dependent variables: local correlation, global correlation), while including site and Y-BOCS scores as regressors in order to identify unique variance associated with PTQ above and beyond variance associated with overall OCD severity. Rigorous significance testing used non-parametric permutation tests (10,000 permutations) implemented by CONN with a whole-brain cluster-level false discovery rate (FDR) correction of *p* < 0.05 (voxelwise threshold of *p* < 0.001). Only those clusters located within an MNI-normalized in-brain mask were retained.

#### Follow-up analyses

To determine the relationship between neural measures related to PTQ and clinical symptoms related to OCD symptom subtypes, we conducted partial correlations between standardized values extracted from significant clusters and the four dimensions of the DOCS (with site specified as the partialing variable). In order to determine whether the relationships between PTQ and connectivity measures were moderated by the presence of Axis I comorbidity or psychotropic medication use in patients, we performed a moderated linear regression predicting connectivity in each cluster that was significantly related to PTQ from the primary analysis (6 clusters for local correlation, see “Results”) using the following mean-centered regressor variables: PTQ score, Axis I comorbidity (yes/no) (see Table S1), psychotropic medication (yes/no) (see Table S2), comorbidity x PTQ, medication x PTQ, Y-BOCS score, and site. A parallel regression was run using BAI and QIDS scores (instead of comorbidity and medication variables) to determine whether state anxiety or depression moderated the relationships between PTQ and connectivity measures.

To aid in interpretation of the findings with regard to connectivity patterns in comparison to HC subjects, we performed a median split on PTQ scores in the OCD group and compared “high-PTQ” OCD patients (*n* = 39, patients greater than the median of 2.33; mean PTQ = 3.06, standard deviation [SD] = 0.39) and “low-PTQ” OCD patients (*n* = 46, patients including and less than the median of 2.33; mean PTQ = 1.62, SD = 0.65) to a sample of HC. Mean standardized values (*z* score) were extracted from each cluster that was significantly associated with PTQ in OCD at whole-brain-corrected significance levels (see “Results”). Standardized values were plotted for each of the 3 groups (high-PTQ OCD, low-PTQ OCD, HC) to determine if OCD patients differ from HC in these measures. Post-hoc testing computed pairwise comparisons between high-PTQ OCD patients and HC, and low-PTQ OCD patients and HC, within a univariate ANOVA (3 levels for group) with site as a covariate (2 dummy variables for 3 levels).

## Results

Demographic and clinical information for the OCD and HC groups are shown in Table 1.

**Table 1:** Demographic and clinical information.

	Controls (*n* = 51)				OCD (*n* = 85)				Group Comparisons
	Mean		*SD*		Mean		*SD*		
Demographics									
Age (years)	32.0		10.8		31.2		10.9		*t*(134) = −0.41, *p* = 0.68
Education (years)	15.9		2.2		15.6		2.0		*t*(134) = −0.82, *p* = 0.42
Biological Sex	25 M/26 F				31 M/54 F				χ^2^(1) = 2.07, *p* = 0.15

	Controls				OCD				
	Mean	*SD*	Range		Mean	*SD*	Range		Group Comparisons
Clinical Information									
PTQ (Mean score)	0.4	0.5	0.0	1.7	2.3	0.9	0.1	3.9	*t*(130.64) = 15.88^, *p* < 0.001
BAI (Mean score)	0.08	0.1	0.0	0.6	0.8	0.5	0.0	2.2	*t*(95.7) = 11.73^, *p* < 0.001
^ #^QIDS (Mean score)	0.2	0.2	0.0	0.6	0.8	0.5	0.0	2.1	*t*(95.7) = 11.73^, *p* < 0.001

					OCD				
OCD Symptoms					Mean	*SD*	Range		
Y-BOCS Total					23.8	5.5	14.0	38.0	
DOCS (Mean score)									
Contamination					1.2	1.1	0.0	3.8	
Responsibility for Harm					1.2	1.0	0.0	4.0	
Unacceptable Thoughts					1.3	1.1	0.0	3.8	
Symmetry/NJREs					1.6	1.0	0.0	4.0	

Each question on the BAI and QIDS received a score between 0 and 3; total scores were calculated as the mean across all answered questions. Each question on the PTQ and DOCS received a score between 0 and 4; total scores were calculated as the mean across all answered questions. Averages (rather than sums) were taken for self-report questionnaires in order to account for any missed responses (<0.001% of all questions). Y-BOCS scores were computed as the sum across all scored questions (none missing), with totals ranging from 0 to 40.

*PTQ* perseverative thinking questionnaire, *BAI* beck anxiety inventory, *QIDS* quick inventory for depressive symptomatology, *DOCS* dimensional obsessive-compulsive scale, *Y-BOCS* yale-brown obsessive compulsive scale, *NJREs* not-just-right experiences, *SD* standard deviation.

^Levene’s test of homogeneity of variance across the groups for age was not assumed, and degrees of freedom were adjusted using Satterthwaite’s approximation.

^#^Only 25 healthy controls and all OCD patients had data on the QIDS.

### PTQ scores

**Fig. 1:**
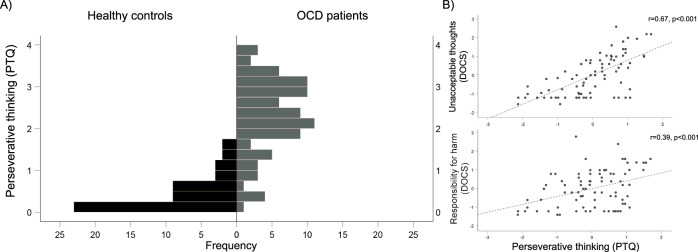
Perseverative thinking scores. **A** Histograms showing the distributions of perseverative thinking questionnaire (PTQ) scores in OCD patients (gray) and healthy controls (black). PTQ scores were calculated as the average of all items on the scale (range: 0–4). **B** Associations between PTQ scores and OCD symptom dimensions as assessed using the Dimensional Obsessive-Compulsive Scale (DOCS) within OCD patients only. Plotted values represent residuals (site was including as a covariate in models).

As would be expected, PTQ scores were significantly higher among OCD patients than HC (F_1,133_ = 157.7, *p* < 0.001, estimated marginal means covarying for site: OCD: 2.29, HC: 0.42). Inspection of the distributions of scores in each group revealed that OCD patients showed a wide range of perseverative thinking scores (range: 0.13 to 3.93; Fig. 1A), with a distribution not significantly different from normal (KS test statistic = 0.81, *p* = 0.20). The HC group, on the other hand, had PTQ scores that were largely at the lower end of the scale (range: 0.00–1.73), with a distribution that was highly positively-skewed (KS test statistic = 0.171, *p* = 0.001; skewness = 1.06).

Within OCD patients, PTQ score was non-significantly positively correlated with overall symptom severity as assessed with the Y-BOCS (*r* = 0.19, *p* = 0.076, using site as covariate). With regard to symptom dimensions, PTQ score was positively correlated with symptoms in the “unacceptable thoughts” and “responsibility for harm” dimensions of the DOCS (using site as covariate; *r* = 0.66 and *r* = 0.39, respectively, both *p* < 0.001) (Fig. 1B). PTQ scores were not related to symptoms in the “contamination” or the “symmetry/NJRE” dimensions. PTQ scores were also significantly positively related to both state anxiety (*r* = 0.57, *p* < 0.001) and depression (*r* = 0.58, *p* < 0.001) (using site as covariate).

### Connectivity measures

#### Local correlation

**Fig. 2:**
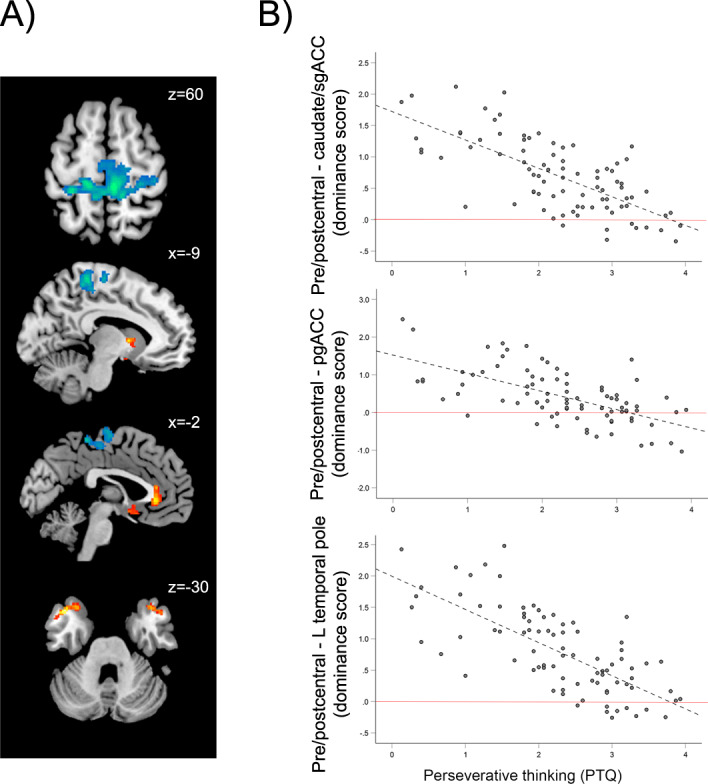
Functional connectivity correlates of perseverative thinking in patients with OCD. **A** Regions where local connectivity was significantly positively (warm colors) and negatively (cool colors) related to perseverative thinking in OCD patients, above and beyond variance associated with overall symptom severity (using the Yale-Brown Obsessive-Compulsive Scale) and site. **B** Within OCD patients, relationships between PTQ scores and “dominance” of local connectivity in pre/postcentral gyrus over caudate/sgACC, pgACC, and left temporal pole clusters. Red lines represent the zero point, where the strength of local connectivity in pre/postcentral gyrus did not differ from local connectivity in the specified regions; points above the red line reflect relative “dominance” of pre/postcentral connectivity over the specified regions. In order to illustrate the high proportion of patients exhibiting pre/postcentral dominance, the plots display raw PTQ scores plotted against direct subtractions of local connectivity values (rather than residuals after accounting for variance explained by overall symptom severity and site). Importantly, the significances of the correlations do not change when using those variables as regressors or not. PTQ perseverative thinking questionnaire, sgACC subgenual anterior cingulate cortex, pgACC pregenual anterior cingulate cortex.

In the primary analysis of the association between PTQ scores and local correlation in OCD (above and beyond variance associated with site and Y-BOCS score), there was a significant positive relationship of perseverative thinking with local connectivity in caudate nucleus and adjacent regions of subgenual ACC (sgACC, Brodmann’s area [BA] 25), pregenual ACC (pgACC, BA 24), bilateral temporal pole (BA 38), and right posterior middle temporal gyrus (MTG; BA 21) (Table 2 and Fig. 2A). There was also a negative relationship with PTQ such that patients with higher perseverative thinking scores had reduced local connectivity in a large cluster including bilateral precentral and postcentral gyri, paracentral lobule, and supplementary motor area (SMA) (BAs 3, 4, 5, 6) (Fig. 2A and Table 2).

**Table 2:** Local connectivity related to perseverative thinking.

	BA	k	x	y	z
Positive correlation with PTQ					
Caudate/sgACC (L)	25	102	−10	6	4
pgACC (B)	24	137	2	32	0
Temporal Pole (L)	38	213	−40	18	−34
Temporal Pole (R)	38	91	48	14	−28
Middle temporal gyrus (R)	21	139	66	−26	−14
Negative correlation with PTQ					
Pre/postcentral gyrus/SMA (B)	3, 4, 5, 6	2365	−18	−34	70

*PTQ* perseverative thinking questionnaire, *sgACC* subgenual anterior cingulate cortex, *pgACC* pregenual anterior cingulate cortex, *SMA* supplementary motor area, *BA* Brodmann’s area, k = cluster extent, *L* left, *R* right, *B* bilateral; xyz coordinates are in MNI space.

Regions listed are significant at cluster-level corrected at *p* < 0.05 (voxelwise *p* < 0.001) using permutation testing.

Inspection of extracted values from the above regions revealed that pre/postcentral local connectivity values, although negatively correlated with PTQ score, were (on average in the group) at least 2 times higher than connectivity in the other identified regions. In order to better characterize this relative “dominance” of local circuit connectivity in pre/postcentral gyri compared to the other regions correlated with PTQ score, we computed difference scores for each subject by subtracting standardized local correlation values (*z* scores) for the 5 regions-of-interest (ROIs) positively associated with PTQ (caudate/sgACC, pgACC, left and right temporal pole, and right MTG) from the pre/postcentral cluster. Between 67% and 88% of OCD patients showed positive values for these subtractions, confirming that, for the majority of patients, local connectivity in pre/postcentral gyrus was higher than that observed in the other regions, regardless of PTQ score. This “dominance” of pre/postcentral connectivity was related to PTQ score, with patients with higher perseverative thinking exhibiting less “dominance” of pre/postcentral gyri connectivity. Another way to conceptualize this finding is that OCD patients with relatively more perseverative thinking showed less of an imbalance of connectivity between pre/postcentral gyrus and other regions compared to OCD patients with comparable disorder severity but relatively less perseverative thinking, who showed more of an imbalance (Fig. 2B, all *r* < −0.65; *p* < 0.001).

#### Global correlation

There were no significant relationships between PTQ score and global correlation at the threshold used.

### Relationships between connectivity and OCD symptom dimensions

**Fig. 3:**
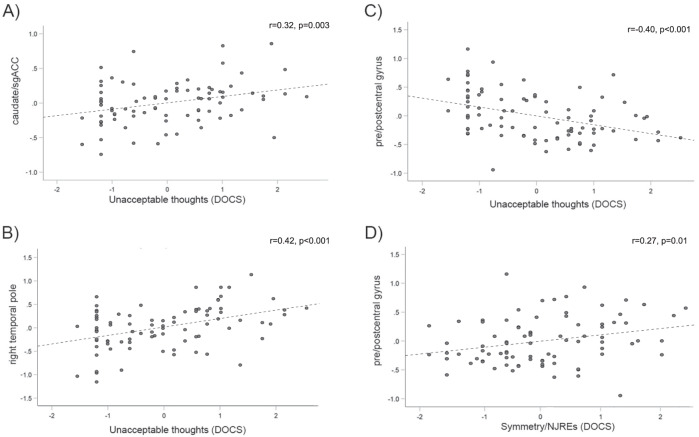
Associations between local connectivity and symptom dimensions as assessed using the dimensional obsessive-compulsive scale (DOCS) in patients with OCD. Plotted values represent residuals for local connectivity (site was included as a covariate in models). sgACC subgenual anterior cingulate cortex, NJREs not-just-right experiences.

Table 3 shows the significant correlations between local connectivity ROIs and DOCS symptom dimensions in the OCD group (site is included as a covariate). The “unacceptable thoughts” symptom dimension was positively related to local connectivity in the caudate/sgACC (Fig. 3A), left and right temporal pole (Fig. 3B), and right MTG (all *r* > 0.27, *p* < 0.01). Local connectivity in pgACC was also related to these symptoms at trend level (*r* = 0.20, *p* = 0.07). Greater pre/postcentral connectivity was associated with reduced severity of this symptom dimension (*r* = −0.40; *p* < 0.001, Fig. 3C), accordingly, greater “dominance” of pre/postcentral connectivity relative to the other regions was related to reduced “unacceptable thoughts” symptoms (all *r* < −0.35, *p* < 0.002).

**Table 3:** Correlations between OCD symptom dimensions and local connectivity.

Symptom Dimension	Significant Correlations with Symptom Dimension			
	Local Connectivity	*r*	Dominance of Pre/postcentral Local Connectivity over	*r*
Unacceptable thoughts	Caudate/sgACC	0.32	Caudate/sgACC	−0.44
	R MTG	0.27	pgACC	−0.35
	L Temporal Pole	0.34	R MTG	−0.40
	R Temporal Pole	0.42	L Temporal Pole	−0.45
	Pre/postcentral	−0.40	R Temporal Pole	−0.51
Symmetry/NJREs	pgACC	−0.21	pgACC	0.28
	R MTG	−0.22	R MTG	0.29
	R Temporal Pole	−0.25	L Temporal Pole	0.29
	Pre/postcentral	0.27	R Temporal Pole	0.32
Responsibility for Harm	Caudate/sgACC	0.23	-	

The effect of site was partialed out of all correlations. Only those correlations significant at *p* ≤ 0.05 are shown. Symptom dimensions were measured using the dimensional obsessive-compulsive scale (DOCS).

*sgACC* subgenual anterior cingulate cortex, *pgACC* pregenual anterior cingulate cortex, *MTG* middle temporal gyrus, *L* left, *R* right, *NJRE* not-just-right experiences.

Interestingly, greater severity of symptoms in the “symmetry/NJRE” dimension of the DOCS was related to higher local connectivity in the pre/postcentral gyrus ROI in OCD patients (*r* = 0.27, *p* = 0.012, Fig. 3D) and greater “dominance” of pre/postcentral local connectivity over other areas (*r* ≥ 0.28, *p* ≤ 0.005 for all ROI comparison variables except pre/postcentral dominance over caudate/sgACC, where *r* = 0.21, *p* = 0.06), as well as reduced local connectivity in pgACC, MTG, and right temporal pole (*r* < −2.1, *p* ≤ 0.05). No clusters were significantly related to the “contamination” symptom dimension, but local connectivity in the caudate/sgACC cluster was positively related to “responsibility for harm” symptoms (*r* = 0.23, *p* = 0.04).

### Effects of psychiatric comorbidity and use of psychotropic medication

PTQ remained a significant predictor of local connectivity (all *t* > 4.1, *p* < 0.001) in the OCD group for the ROIs identified in the primary analysis (sgACC/caudate, pgACC, left and right temporal pole, right MTG, and pre/postcentral gyrus) when including additional regressors accounting for variance related to the presence of Axis I comorbidity and use of psychotropic medication. There were no interactions between PTQ and comorbidity or PTQ and medication for any of the regions investigated.

### Effects of state anxiety and depression

PTQ remained a significantly predictor of local connectivity (all *t* > 2.7, *p* < 0.01) in the OCD group for the ROIs identified in the primary analysis when including additional regressors accounting for variance related to state anxiety (BAI score) and depression (QIDS score). There was a significant interaction between PTQ and BAI scores for the pgACC cluster (*t* = 2.1, *p* = 0.04), reflecting a steeper positive slope between PTQ score and local connectivity in the pgACC among patients with higher BAI scores. An interaction in the opposite direction was found for the right temporal pole cluster (*t* = −2.4, *p* = 0.02), with a steeper positive slope between PTQ score and local connectivity in right temporal pole among patients with lower BAI scores. Finally, there was a significant interaction between PTQ and QIDS scores for the pre/postcentral gyrus cluster (*t* = −2.2, *p* = 0.03), reflecting a steeper negative slope between PTQ and pre/postcentral local connectivity among patients with higher QIDS scores. No other interactions between PTQ and BAI or PTQ and QIDS were found.

### Comparison with healthy controls

**Fig. 4:**
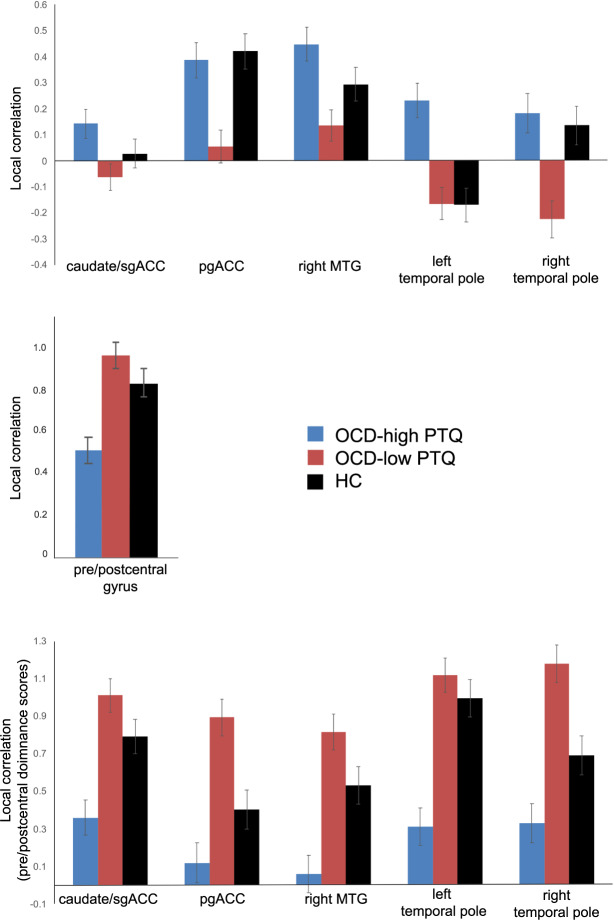
Average local connectivity for OCD patients with “high” perseverative thinking (PTQ > median of the OCD group) (blue), OCD patients with “low” perseverative thinking (PTQ ≤ median of the OCD group) (red), and healthy controls (HC) (black). In the bottom figure, pre/postcentral dominance scores reflect connectivity values for pre/postcentral gyrus minus those for the other regions listed. All plotted connectivity values are within-subject normalized estimated marginal means (site was included as a covariate in models).

For several ROIs, healthy control subjects showed a pattern of local connectivity that was numerically in-between the high- and low-PTQ OCD groups for the majority of ROIs (Fig. 4 and Table 4). There was more pre/postcentral connectivity “dominance” for HCs than for high-PTQ OCD patients (Fig. 4 and Table 4, bottom panels). However, pre/postcentral “dominance” was numerically smaller for HCs than for low-PTQ OCD patients, who showed the greatest imbalance between pre/postcentral connectivity and the other regions out of the three groups (Fig. 4 and Table 4). This general pattern (where high-PTQ OCD < HC < low-PTQ OCD or high-PTQ OCD > HC > low-PTQ OCD) was found for the majority of ROIs examined except for pgACC and right temporal pole (where estimated marginal means [site as covariate] for the high-PTQ OCD group were similar to HC) and left temporal pole (where the estimated marginal mean [site as covariate] for the low-PTQ OCD group was similar to HC) (Fig. 4 and Table 4; see Supplementary Fig. 1 for group means without site as a covariate).

**Table 4:** Local connectivity in healthy controls compared to low-PTQ OCD patients and high-PTQ OCD patients.

	OCD_Low_PTQ_ (*n* = 46)		Controls (*n* = 51)		OCD_High_PTQ_ (*n* = 39)		
	*EMM*	*SE*	*EMM*	*SE*	*EMM*	*SE*	
Local Connectivity (Beta Values)							
Caudate/sgACC	−0.06	0.05	0.03	0.05	0.14	0.06	
pgACC	0.06	0.06	0.42	0.07	0.39	0.07	
R Middle Temporal Gyrus	0.13	0.06	0.29	0.06	0.45	0.07	OCD_Low_PTQ_ vs. Controls: *p* < 0.001
L Temporal Pole	−0.17	0.06	−0.17	0.07	0.23	0.07	OCD_High_PTQ_ vs. Controls: *p* < 0.001
R Temporal Pole	−0.23	0.07	0.13	0.07	0.18	0.07	OCD_Low_PTQ_ vs. Controls: *p* = 0.002
Pre/postcentral Gyrus	0.95	0.06	0.82	0.06	0.50	0.06	OCD_High_PTQ_ vs. Controls: *p* = 0.002
Dominance of Pre/postcentral Local Connectivity (Beta Values)							
Caudate/sgACC	1.01	0.09	0.79	0.09	0.36	0.09	OCD_High_PTQ_ vs. Controls: *p* = 0.003
pgACC	0.89	0.10	0.40	0.11	0.12	0.11	OCD_Low_PTQ_ vs. Controls: *p* = 0.002
R Middle Temporal Gyrus	0.81	0.09	0.53	0.10	0.06	0.10	OCD_High_PTQ_ vs. Controls: *p* = 0.003
L Temporal Pole	1.12	0.09	0.99	0.10	0.27	0.10	OCD_High_PTQ_ vs. Controls: *p* < 0.001
R Temporal Pole	1.18	0.10	0.69	0.10	0.32	0.11	OCD_Low_PTQ_ vs. Controls: *p* = 0.002
							OCD_High_PTQ_ vs. Controls: *p* = 0.03

Site was used as a covariate in the models. Not shown are the direct comparisons between OCD_High_PTQ_ and OCD_Low-PTQ_ (significantly different for all ROIs), due to the circular nature of such an analysis.

*EMM* estimated marginal means, *SE* standard error of the estimated marginal means, *L* left, *R* right, *sgACC* subgenual anterior cingulate cortex, *pgACC* pregenual anterior cingulate cortex.

## Discussion

The present study investigated individual variability of perseverative thinking in a large OCD sample, linking scores on the PTQ to OCD symptom dimensions and patterns of resting-state functional connectivity. Though obsessions have been characterized as examples of perseverative thinking [14], results identified notable variability in the frequency of PT in our OCD sample, with some patients reporting very high scores of PT and others showing scores that overlapped with healthy controls. This variability likely reflects the variety of psychological phenomena subsumed under the umbrella of “obsessions”, which include not only thoughts, but also urges, sensations, and images, i.e., all the antecedents of compulsions in OCD. The frequency of PT within OCD was related to more symptoms in the “unacceptable thoughts” domain, greater local connectivity in caudate nucleus, sgACC, pgACC, temporal pole, and MTG, and reduced local connectivity in pre/postcentral gyri. These findings remained significant even after accounting for variance associated with general anxiety and depression, the presence of Axis I comorbidities, and psychotropic medication use in our sample.

Our findings linking perseverative thinking to “unacceptable thoughts” and “responsibility for harm” symptoms in OCD confirm and extend a prior study that identified a relationship between “unacceptable thoughts” symptoms and depression-focused rumination (as assessed by the Ruminative Response Scale [RRS]) in a transdiagnostic clinical sample comprised mostly of non-OCD anxious and depressed patients [31]. The “unacceptable thoughts” domain in OCD encompass a range of intrusive, ego-dystonic, and highly distressing thoughts related to violence, sexuality, morality, and religion [13, 73]. These types of symptoms are sometimes referred to as “pure obsessions” because they are associated more with mental (rather than physical) compulsions compared to other OCD symptom dimensions [13, 73]. Our data suggest that PT may be an important treatment target for patients with these types of OCD symptoms.

Neurobiologically, local connectivity within the caudate, subgenual and pregenual anterior cingulate cortex, bilateral temporal pole, and posterior middle temporal gyrus was associated with higher PT within the OCD sample. The temporal poles have been linked to many higher-order cognitive-affective functions, including semantic representation, autobiographical memory, and social cognition/theory of mind [74]. They are part of the DMN (but see further discussion below), which, together with other regions in the temporal lobe (middle temporal gyrus, parahippocampus), medial prefrontal cortex, posterior cingulate, and posterior inferior parietal lobe, have been linked to a variety of “internally-focused” processes such as thinking about the self, autobiographical memory, and simulating and planning future events [36–38]. The pregenual ACC area that was correlated with PT in this study overlaps with the “anterior medial prefrontal cortex” node identified by Andrews-Hanna and colleagues [75] as a core component of DMN. Subgenual ACC, although not always considered a canonical part of DMN, has been associated with negative affect [76–80] and harm avoidance [81], and is more functionally connected to DMN in association with higher rumination in depression [41, 47, 82, 83]. Importantly, the present relationships of DMN connectivity with PTQ were not merely accounted for by the higher levels of general anxiety and depression in the OCD sample. When considered alongside prior studies linking DMN to perseverative cognition in major depression [40–49, 82, 84], and generalized anxiety [85–87], the present findings suggest that altered DMN functioning may be a transdiagnostic mechanism of PT. Future studies should test this hypothesis directly because of the clinical implications: if the neurobiology of PT is transdiagnostic, then treatments targeting PT should be as well; conversely, if the neural substrates of PT differ across disorders, more disease-specific therapies will be needed.

It should be noted that the temporal pole contributions to DMN are less well characterized than the “core” regions of medial frontal and parietal cortex [88]. In a 7-network parcellation computed by Yeo and colleagues [89] from 1000 individuals, the superior-lateral aspects of the temporal poles are part of a DMN parcellation while inferior-medial regions are assigned to a “limbic” network that also includes orbitofrontal cortex. An examination of the overlap between the temporal pole clusters identified in the present study and Yeo et al.’s 7-network parcellation revealed that approximately half of the voxels in each hemisphere were located in the DMN parcellation and half were in the limbic network. Although these results should be interpreted with caution—while the majority of DMN parcellations in the literature assign some part of the temporal poles to DMN, there appears to be variability across studies in the assignment of specific voxels within the temporal pole area [88]—these data suggest that our temporal pole findings may be in “transition zones” bridging between networks involved in internally-focused cognitive states (i.e., DMN) and emotion processing (i.e., limbic areas).

Unexpectedly, greater PT in OCD was associated with relatively less local connectivity in a large bilateral cortical cluster encompassing precentral and postcentral gyri, and extending into paracentral lobule and SMA, areas involved in somatosensory and motor processing [90]. Prior work indicates that, among healthy individuals, sensorimotor cortical areas are tightly functionally connected at rest, exhibiting higher local connectivity than many other areas of the brain [91]. Our data is consistent with this organizational principle, with the majority of OCD patients showing higher local connectivity in sensorimotor regions compared to the other areas that were related to PT. Indeed, this relative “dominance” (or imbalance) of sensorimotor connectivity was associated with PT such that OCD patients with higher PT showed less sensorimotor dominance and patients with lower PT showed more sensorimotor dominance. Interestingly, sensorimotor dominance was related to OCD symptom dimensions such that greater dominance was associated with more “symmetry/NJRE” symptoms among OCD patients, whereas lesser sensorimotor dominance was associated with more “unacceptable thoughts” symptoms. Furthermore, when comparing OCD patients with a group of HCs, HCs exhibited a pattern of sensorimotor dominance that was intermediate to OCD patients with higher and lower PT. Put together, these findings suggest a u-shaped function whereby “too much” or “too little” sensorimotor connectivity dominance may be linked to different types of symptoms in OCD. Conio and colleagues [92] recently argued that the relative dominance of sensorimotor to default mode network activity could reflect underlying differences in serotonergic and dopaminergic neurotransmission. Specifically, they suggest that either increases in serotonin, or decreases in dopamine signaling, should result in a shift away from sensorimotor and towards default mode dominance resulting in less attention to external stimuli and more internally-directed thought (such as PT). Although the present work cannot directly speak to this question, future studies should further explore this intriguing idea by testing the relationships between neurotransmitter levels, behavior, and intrinsic network dominance in OCD.

Prior work examining group differences between OCD and controls in local connectivity, often in the form of analysis of regional homogeneity (“ReHo”), have pointed to some of the same regions as those identified here. A meta-analysis conducted by Hao and colleagues [93] identified lower ReHo in OCD compared to controls in an area that overlaps with the sensorimotor region we identified as showing a negative relationship with PT. This meta-analysis also found decreased ReHo for OCD in the left caudate, an area that was instead positively correlated with PT in our study. The present work complements and extends these prior findings, suggesting that some differences identified in prior group comparisons may also be related to symptom heterogeneity. This work also highlights the fact that even within a given disorder, the particular symptom constellations present in the sample can influence the identified neural correlates. These symptom factors should ideally be considered as a moderating factor in future group comparisons involving heterogenous disorders.

There are several limitations to this work. The main study analysis examining the relationship between PT and connectivity in OCD used data acquired across two different MRI scanners. Although we harmonized the acquisition sequences as much as possible (given hardware limitations), used identical processing streams on all the data, and used site as a covariate in group models in accordance with suggestions for handling site-based clustering effects [63] and the procedures of large-scale multi-site studies [64, 65], there could be subtle residual differences for which we have not accounted. Further, because we felt it was critical to include patients with certain comorbidities and those taking psychotropic medications for their OCD in order to increase the representativeness of our findings and applicability to the general population of OCD patients (where comorbidities and the use of medications are extremely common), a majority of the patients had comorbidities and/or were taking psychotropic medications that could affect behavior and brain function. Critically, relationships between local connectivity and PTQ scores remained significant even after accounting for variance associated with psychiatric comorbidity and medication use, indicating that these factors were not driving the reported findings. Finally, the measurement of PT used in this study was self-report. Although PT, by its very nature, is an internal, personal experience that is not readily observable, insight into one’s own mental processes can vary and bias self-report responses. Although the reliance on a patient’s report of their internal experiences is common among gold-standard methods for ascertaining symptoms in psychiatry and psychology, future work should seek to further develop and implement objective behavioral tasks to measure PT. To this end, we have previously reported on the use of a task-switching paradigm in a separate, smaller cohort of OCD patients, which found that patients made more errors than matched controls when performing an externally-focused cognitive task (target detection) when it followed a period of thinking about a personal negative future events [39], a finding that could be interpreted as OCD patients displaying a relative failure to adequately disengage from internal, negative thought. Other investigators have also developed tasks to try to index internally-focused attention behaviorally, focusing on reaction time or pupil diameter as a proxy for PT [23, 86, 94, 95]. Although, unfortunately, our task-switching paradigm [39] was not used in the same cohort of patients as reported in the present study, future work should aim to conduct both self-report and behavioral assessments of PT in the same participants in order to evaluate the relationship between measures. Despite these limitations, the present study is the first to identify clinical and neural correlates of PT in OCD. Results identifying greater connectivity of DMN areas and reduced dominance of sensorimotor connectivity among patients with higher PT reinforce the importance of considering symptom heterogeneity when seeking biomarkers of OCD. Further, the findings suggest that treatments known to alter the balance between DMN and other intrinsic brain networks and enhance the capacity to attend to external stimuli (such as mindfulness-based therapies [96] or drugs enhancing dopaminergic neurotransmission [92]) may be helpful in targeting PT. Further study of the neural correlates of PT in large transdiagnostic samples could inform the development of such novel interventions within or across disorders.

## Supplementary information

Supplemental Information
