# Supplementary material for: Imbalance between default mode and sensorimotor connectivity is associated with perseverative thinking in obsessive-compulsive disorder
Source: Transl Psychiatry. 2022 Jan 12;12:19. doi: 10.1038/s41398-022-01780-w (PMC8755709; doi:10.1038/s41398-022-01780-w)
Supplement: Supplementary file 1 — Supplemental Information [file 41398_2022_1780_MOESM1_ESM.docx]

Imbalance between default mode and sensorimotor connectivity is associated with perseverative thinking in OCD

Supplemental Materials and Methods

Comorbidities and Medications

Twenty-eight out of 85 OCD patients (33%) had no Axis 1 comorbidities (Table S1); the remaining 57 patients (67%) had at least one current comorbid Axis I disorder including generalized anxiety disorder (n=31), panic disorder (n=16), excoriation disorder (n=11), attention deficit hyperactivity disorder (n=11), social anxiety disorder (n=10), body dysmorphic disorder (n=9), and agoraphobia (n=8). Less frequent current comorbidities included illness anxiety disorder (n=5), alcohol use disorder (mild, n=5), trichotillomania (n=4), hoarding disorder (n=4), Tourette’s disorder (n=3), substance use disorder (mild, n=3), binge eating disorder (mild, n=3; moderate, n=1), post-traumatic stress disorder (n=3), major depressive disorder (n=3), persistent tic disorder (n=2), somatic symptom disorder (n=2), anorexia nervosa (n=1), and bulimia nervosa (mild, n=1). Forty-one of the 85 patients (48%) were not taking psychotropic medications (Table S2); the remaining 44 patients (52%) were taking antidepressants targeting monoaminergic neurotransmission (including serotonin reuptake inhibitors, serotonin modulator and stimulators, and tricyclic antidepressants) (n=39), benzodiazepines as needed (n=10), atypical antipsychotics (n=5), anticonvulsants (n=4), stimulants (n=3), bupropion (n=3) and psychoactive antihypertensives (n=2). Table S2 lists the specific medications being taken in the OCD group.

Neuroimaging data acquisition and preprocessing

MRI scanning occurred on Siemens 3T scanners. NYUSoM- and NKI-recruited participants were scanned on a MAGNETOM TrioTim and ISMMS-recruited participants were scanned on a MAGNETOM Skyra using a 32-channel head coil, with sequences harmonized between the scanners. Although all healthy controls recruited at ISMMS were scanned on the same scanner, sequences with minor differences were used for controls scanned before and after 2017. All MRI scanning sequences for ISMMS-recruited OCD patients were identical to the sequences utilized for ISMMS-recruited controls after 2017.

Structural data were obtained using a T1-weighted MP-RAGE protocol at both ISMMS (before and after 2017) and NKI (repetition time [TR]=2400ms, flip angle=8°, field-of-view [FOV]=256mm, 0.80mm isotropic voxels). For the MP-RAGE, the orientation of acquisition, echo time (TE), and number of slices were different between the two scanning sites (ISMMS before and after 2017: TE=2.07ms, 224 slices, in the oblique orientation for acquisition before 2017, and transverse orientation after 2017; NKI: TE=2.01ms, 208 slices, sagittal acquisition). Resting-state fMRI data were acquired using a high-resolution multiband-accelerated echo-planar sequence for full brain coverage (TR=1000ms, flip angle=60°, FOV=228mm, 2.1mm isotropic voxels, no gap). For the two sequences used in the OCD sample, the echo time was slightly different (ISMMS TE=25ms; NKI TE=25.4ms). For the sequence used for healthy controls before 2017 at ISMMS, echo time, the number of slices, and acceleration factor differed (ISMMS before 2017: 70 slices, acceleration factor=7, TE=35ms; ISMMS after 2017 and NKI: 72 slices, acceleration factor: 6). As described in the manuscript, site was used as a covariate in all analyses.

Within-subject normalization

The use of within-subject normalization during denoising allows for the identification of localized differences in connectivity patterns while increasing signal-to-noise by removing any potential global (brain-wide) differences between subjects, which can be artifactual (such as residual motion-related effects). However, there could also be meaningful, non-artifactual global connectivity differences between subjects that relate to PTQ score, which we would not be able to detect when using within-subject normalization. In order to address this issue, we re-ran the main analyses without using within-subject normalization. No new (global or other) effects (for local or global correlation measures) were identified in relation to PTQ score. For local correlation, the pgACC, left temporal pole, and pre/postcentral gyri clusters were still significantly related to PTQ score at the current threshold (cluster-level whole-brain corrected to p<0.05 with a voxelwise threshold of p<0.001 using permutation analysis). The other clusters – caudate/subgenual ACC, right temporal pole, and right MTG – were identified at a slightly higher p-value threshold (cluster-level whole-brain corrected to p<0.05 with a voxelwise threshold of p<0.005 using permutation analysis). These data suggest that the use of within-subject normalization increased signal-to-noise without masking global differences of interest, and that relationships between PT and connectivity were localized to specific brain areas.

Relationship between connectivity measures and PTQ score without including Y-BOCS as covariate

To identify relationships between connectivity and PTQ score above and beyond connectivity related to general OCD severity, we included Y-BOCS total scores as a covariate (regressor) in the primary regression model. PTQ score was only weakly positively correlated with Y-BOCS score (r=0.19, p=0.079). In order to evaluate the impact of including Y-BOCS as a covariate, we conducted follow-up partial correlations examining the association between the 6 clusters identified in the local correlation analysis and PTQ score, omitting Y-BOCS as a variable in the model and only including site as the partialed covariate. All reported clusters remained significantly related to PTQ score (all r>0.46 [positive correlations] and r<-0.62 [negative correlations], all p<0.001) when not including Y-BOCS as a covariate.

Supplemental Figure 1. Average local connectivity for OCD patients with “high” perseverative thinking (PTQ>median of the OCD group) (blue), OCD patients with “low” perseverative thinking (PTQ≤median of the OCD group) (red), and healthy controls (HC) (black). In the bottom figure, pre/postcentral dominance scores reflect connectivity values for pre/postcentral gyrus minus those for the other regions listed. All plotted connectivity values are within-subject normalized means with no covariates.

Table S1: Clinical comorbidity for OCD patients (*n* = 85).

Table S2: Medication information for OCD patients (*n* = 85).

^ Antidepressants targeting monoaminergic neurotransmission included serotonin reuptake inhibitors (including SSRI, SNRI), serotonin modulator and stimulators, and tricyclic antidepressants. SSRI = Selective Serotonin Reuptake Inhibitor; SNRI = Serotonin-Norepinephrine Reuptake Inhibitor.
